# Interaction of serum zinc and copper status with fatty acid desaturases on incidence of type 2 diabetes in the EPIC-Potsdam study

**DOI:** 10.1016/j.redox.2025.103998

**Published:** 2025-12-26

**Authors:** Marcela Prada, Olha Bezuhla, Olga Kuxhaus, Fabian Eichelmann, Susanne Jäger, Anna P. Kipp, Hajo Haase, Tanja Schwerdtle, Matthias B. Schulze

**Affiliations:** a Department of Molecular Epidemiology, German Institute of Human Nutrition Potsdam-Rehbruecke, Nuthetal, Germany; b German Center for Diabetes Research (DZD), München-Neuherberg, Germany; c Department of Nutritional Physiology, Institute of Nutritional Sciences, Friedrich Schiller University Jena, Germany; d Department of Food Chemistry and Toxicology, Technische Universität Berlin, Berlin, Germany; e Institute of Nutritional Science, University of Potsdam, Nuthetal, Germany; f Max Rubner-Institut (MRI), Bundesforschungsinstitut für Ernährung und Lebensmittel, Karlsruhe, Germany

**Keywords:** Copper, Desaturases, Diabetes, Fatty acids, Genes, Zinc

## Abstract

**Aims:**

To examine whether zinc (Zn) and copper (Cu) status influence the association of estimated delta-5 desaturase (D5D), delta-6 desaturase (D6D), and stearoyl-CoA desaturase-1 (SCD1) activities with type 2 diabetes (T2D) risk.

**Methods:**

We used a nested case-cohort design within the EPIC–Potsdam Study (n = 1979; 447 incident T2D cases). Desaturase activities were estimated using erythrocyte fatty acids (FA): D5D (20:4n-6/20:3n-6), D6D (18:3n-6/18:2n-6), and SCD1 (16:1/16:0 [SCD1-16], 18:1/18:0 [SCD1-18]). We evaluated associations between desaturases and serum Zn or Cu, assessed interactions between serum Zn or Cu and desaturase activities in Cox regression models for T2D risk, and examined modification by Zn transporter *SLC30A8* genetic variant and metal-related polygenic risk scores.

**Results:**

Higher serum Zn was significantly associated with lower SCD1-18 activity (β per 1 SD = −0.09). Zn status showed a non-linear modifying effect on the D5D-T2D relationship (p-interaction = 0.03), though an inverse D5D association was observable consistently across Zn levels. Serum Cu was positively associated with SCD1-16 (β = 0.13) and SCD1-18 (β = 0.08) and negatively associated with D5D activity (β = −0.13). Stronger inverse associations of higher D5D activity with T2D risk were observed at low Cu levels (HR 0.69, 95% CI 0.58–0.81) versus higher levels (HR 0.95, 95% CI 0.80–1.13) (p-interaction = 0.009). The *SLC30A8* variant rs13266634 significantly modified the D5D-T2D association. Furthermore, the inverse association of D5D with T2D was stronger among participants with a higher Cu genetic score.

**Conclusions:**

Zn and Cu status modified the relationship between FA desaturases and T2D risk. This was supported by serum Zn and Cu levels and by genetic variation related to their transport and homeostasis.

## Graphical abstract

**Figure:**
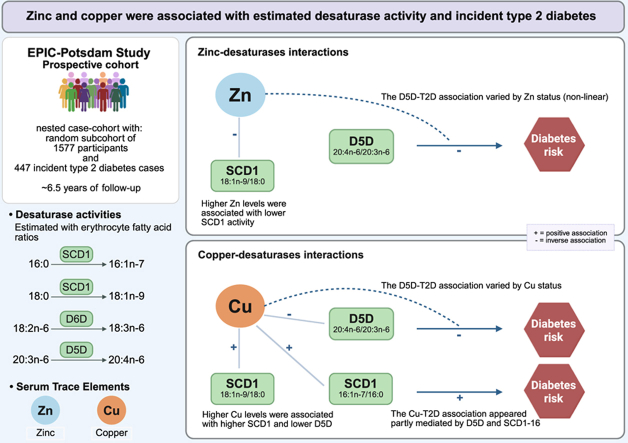

**Table:** ABBREVIATIONS

Cu	Copper
D5D	Delta-5 desaturase
D6D	Delta-6 desaturase
EPIC	European Prospective Investigation into Cancer and Nutrition
FA	Fatty acid
Fe	Iron
GWAS	Genome-wide association studies
PGS	Polygenic risk score
SCD1	Stearoyl-coA desaturase-1
SCD1-16	Ratio of palmitoleic acid/palmitic acid (16:1n-7/16:0)
SCD1-18	Ratio of oleic acid/stearic acid (18:1n-9/18:0).
*SLC30A8*	Zinc transporter 8 gene
SNP	Single-nucleotide polymorphisms
Zn	Zinc

## 1 Introduction

Fatty acid desaturases are a family of enzymes that introduce double bonds into fatty acid (FA) chains. Delta-5 desaturase (D5D) and delta-6 desaturase (D6D) synthesize long-chain polyunsaturated FA [1], while stearoyl-CoA desaturase-1 (SCD1), a delta-9 desaturase, converts saturated FA into monounsaturated FA. Epidemiological studies have linked a higher estimated D6D activity with a higher risk of developing type 2 diabetes (T2D), whereas higher D5D activity appeared to have a protective effect [[2], [3], [4], [5], [6]]. Furthermore, higher estimated SCD1 activity has been associated with higher risk of incident T2D [3,7]. However, recent evidence points toward SCD1 activity having protective or harmful effects depending on the tissue and metabolic context [8,9].

Given the importance of desaturase enzymes in FA metabolism and their association with T2D, it is crucial to consider factors that influence their activity. In male Wistar rats, zinc (Zn) deficiency has been shown to alter the activity of desaturase enzymes [10]. Zn also appears relevant to T2D in humans: higher serum Zn concentrations were linked to an increased T2D risk [11], a finding we also observed in the EPIC-Potsdam Study [12]. Previous observational research indicated that serum Zn concentrations were associated with serum n-6 polyunsaturated FA concentrations and estimated D5D and D6D activities in men, and that gamma-linoleic acid (18:3n-6) was positively associated with T2D incidence only among those with higher serum Zn concentrations [13]. Additionally, the associations of D6D and linoleic acid (18:1n-6) with the metabolic syndrome were stronger among those with a higher serum Zn concentration [14]. These findings suggest that serum Zn concentrations may modify the relationship between polyunsaturated FA metabolism and T2D risk.

Copper (Cu) has been linked to lipid metabolism by influencing desaturase activities [15]. In male rats, Cu deficient diets altered D5D and SCD1 activities [16,17]. In humans, a low-Cu diet led to decreased serum oleic acid (18:1n-9) and increased arachidonic acid (20:4n-6) levels [18]; however, evidence from human studies remains limited. Despite these observations, the potential influence of Cu on the association between desaturase activities and incident T2D in humans is unexplored. In addition to Zn and Cu, iron (Fe) is another metal potentially relevant to desaturation, since SCD1 requires iron for its catalytic function [19]. However, in contrast to Zn and Cu, there is less evidence from human studies that Fe directly modulates desaturase activities.

Beyond circulating biomarkers, genetic variation in metal transport and homeostasis may offer insights into how Zn and Cu relate with FA desaturation and diabetes risk. Genome-wide studies (GWAS) have identified single nucleotide polymorphisms (SNPs) associated with circulating Cu and Zn [[20], [21], [22], [23]]. Furthermore, of particular mechanistic relevance is rs13266634 in the *SLC30A8* gene, which encodes Zn transporter-8 (ZnT8), a β-cell-specific Zn transporter involved in glucose-stimulated insulin secretion [24]. This variant is one of the most consistently replicated genetic markers for T2D in GWAS and meta-analyses [25,26] and was suggested to modify the relationship between plasma Zn and T2D [27]. Combining GWAS-identified metal variants with the β-cell Zn transporter variant allows to examine tissue-level Cu and Zn effects on desaturase pathways and T2D risk, adding mechanistic context to observational associations.

With data from a prospective cohort study, we first aimed to examine the associations of serum Cu and Zn with circulating FA profiles and estimated activities of SCD1, D5D, and D6D. To determine whether trace elements modify the desaturase-diabetes relationships, in a second step we investigated interactions between serum Cu and Zn and desaturase activities on T2D risk. We then conducted mediation analyses to determine whether desaturase activities mediate the relationships between Zn or Cu and T2D. We also investigated whether genetic variants related to Zn or Cu modify the associations of the desaturases and related FAs with T2D risk.

## 2 Research design and methods

### 2.1 Study population

The European Prospective Investigation into Cancer and Nutrition (EPIC)-Potsdam study includes 27548 individuals (16,644 women aged mainly 35–65 years and 10904 men aged mainly 40–65 years) from the general population of Potsdam, Germany, recruited between 1994 and 1998 [28]. Informed consent was obtained from all participants, and approval was given by the Ethical Committee of the State of Brandenburg, Germany. The baseline assessment included blood collection, anthropometry, a questionnaire addressing lifestyle and socio-demographic characteristics, and an interview on prevalent diseases [29,30]. Follow-up questionnaires were administered every 2–3 years to identify new cases of T2D. Participation rates for four follow-up rounds were 90%–96% and extended up to 2005.

To enhance molecular phenotyping efficiency, a case-cohort was implemented in the EPIC-Potsdam study. From individuals with blood samples (n = 26437), a random subcohort was selected (n = 2500), and every case with newly developed diabetes was identified during an average of 6.5 years of follow-up (n = 801) (Fig. S1). We excluded individuals due to lack of Zn and Cu measurements (n = 297), unreliable FA measurements (n = 833), missing follow-up information (n = 36), prevalent or non-verifiable cases (n = 72), and missing covariables (n = 10). The final analytical sample comprised 1979 individuals: 1577 from the subcohort and 447 incident diabetes cases, with 45 participants overlapping.

### 2.2 Measurements of Cu, Zn, Fe, and FA

From blood collected at baseline, erythrocytes, buffy coat, serum, and plasma were preserved at −80 °C or lower temperature. The laboratory procedure for trace element profiling has been described before [12]. In short, 50 μL of the serum sample was mixed with 440 μl of diluent solution. As internal standard and for isotope dilution analysis, a solution of 10 μL having 50 μg/L^77^Se and 5 μg/L Rh was added, resulting in a total volume of 500 μL. The solution was analysed via inductively coupled plasma mass spectrometry (ICP-MS/MS) (Agilent ICP-QQQ-MS 8800, Agilent Technologies, Waldbronn, Germany). For external calibration, standards were prepared matrix-matched in the diluent solution. For quality control, reference material RECIPE® ClinChek® serum control lyophilized (Ref. 8880–8882, Lot 347 or Lot 1497) was assessed in triplicate daily. The mean recoveries were: Zn 96.9% ± 6.1%; Cu 95.5% ± 6.8%; Fe: 100.2% ± 7.0%. Additionally, enough blank samples (distilled H_2_O) were included daily to determine limits of detection (3ϭ-criterion) and quantification (10ϭ-criterion).

The FA of red blood cell membranes were analysed by gas chromatography and expressed as the percentage of the total FA identified in the chromatogram, which included thirty-two FA [31]. We calculated FA ratios to reflect estimated desaturase activities. D5D activity was estimated as the ratio arachidonic acid/dihomo-gamma-linolenic acid (20:4n-6/20:3n-6), and D6D activity as the ratio gamma-linoleic acid/linoleic acid (18:3n-6/18:2n-6). SCD1 activity was estimated in two ways: as the ratio of palmitoleic acid/palmitic acid (SCD1-16, 16:1n-7/16:0) and as the ratio of oleic acid/stearic acid (SCD1-18, 18:1n-9/18:0).

### 2.3 Genotyping and SNP selection

DNA was extracted from buffy coats using the chemagic DNA Buffy Coat Kit special on a Chemagic Magnetic Separation Module I (PerkinElmer Chemagen technologies, Baesweiler, Germany), according to the manufacturer's manual. Afterwards, three genotyping arrays were used for the genotyping of samples: HumanCoreExome-12v1-0_B (n = 675, two datasets), Human660W-Quad_v1_A (n = 363), and Illumina InfiniumOmniExpressExome-8v1-3_A DNA Analysis BeadChip (n = 1377). Quality control and genotyping were described elsewhere for the HumanCoreExome-12v1-0_B chips and Human660W-Quad_v1_A [32], and for the Illumina InfiniumOmniExpressExome-8v1-3_A DNA Analysis BeadChip [33]. Imputation and phasing were conducted using the Michigan Imputation Service [34] and pre-phasing using Eagle2 [35]. The Haplotype Reference Consortium was used [36]. Imputation was performed according to the four batches (two for the HumanCoreExome-12v1-0_B chip or one for each chip) utilizing minimac3 [34]. Imputed files were merged retaining the lowest R^2^ score among all four files. Then, SNPs were screened by R^2^ with a criterion to retain those exhibiting values greater than 0.6. Genotypic data from 1890 case-cohort samples were accessible.

We identified SNPs related to circulating Zn and Cu levels from published GWAS [[20], [21], [22], [23]] available in our dataset (**Supplementary Methods**). The Cu-related candidates were rs2769264, rs17564336, rs1175550, rs3858704, rs10424895, rs34004251. The Zn-related candidates were rs2120019, rs1532423, rs322884. In addition, we prespecified rs13266634 in *SLC30A8* as a variant involved in Zn transport and with strong prior evidence for T2D (Supplementary Table 1).

### 2.4 Assessment of type 2 diabetes

Incident T2D was identified from self-reported medical diagnoses, medication, and dietary treatment. Furthermore, additional information from death certificates or from sources such as tumor centers, physicians, or clinics was obtained. For participants who were classified as potential cases based on that information, a standard inquiry form was sent to the treating physician. Only cases with a diagnosis of T2D verified by a physician (according to the International Classification of Diseases, 10th Edition: E11) and a diagnosis date after the baseline investigation were considered incident cases of T2D.

### 2.5 Other measurements

Information on education, smoking habits, physical activity (both leisure-time and occupational), alcohol intake, medical history, and other relevant covariates was gathered at baseline using self-administered questionnaires and a computer-guided interview. The average time spent on sports activities, gardening, and biking in the 12 months prior to baseline was considered as leisure-time physical activity. Anthropometric measurements (waist circumference and height) were conducted using standard protocols with strict quality control [29]. Plasma total and HDL-cholesterol and triglycerides were measured as described before [37].

### 2.6 Statistical analysis

To investigate the relation of FA levels or desaturase ratios with Zn, Cu, and Fe, we conducted linear regression analyses with adjustment for potential confounders. Exposures and outcomes were log-transformed to normalize distributions and then Z-standardized (mean = 0, SD = 1) using subcohort-specific parameters, resulting in standardized effect estimates.

Associations between FA and diabetes risk were evaluated using Cox regression models for the case-cohort design according to the Prentice method [38]. FA exposures were log-transformed and Z-standardized (mean = 0, SD = 1), hence, estimates correspond to a 1-SD increment. Age was used as the underlying time scale, with entry time defined as the participant's age at recruitment and exit time defined as the age at the end of follow-up (date of death, diagnosis, or return of the last follow-up questionnaire). Models were stratified by age and adjusted for sex, waist circumference (continuous), height (continuous), smoking status (never, past, current < 20 cigarettes/day, current ≥ 20 cigarettes/day), leisure-time physical activity (hours/week), occupational activity (light, moderate, heavy), education (in or no training, skilled worker, technical school, or university degree), alcohol intake (0, 0.1–5.0, 5.1–10.0, 10.1–20.0, 20.1–40.0, or >40.0 g/day), and fasting status. In additional models, we further adjusted for lipid markers (total cholesterol, HDL-cholesterol, and triglycerides), medication (antihypertensive, lipid-lowering, and acetylsalicylic acid), and serum Cu, Zn or Fe levels. To account for potential nonlinearity in associations with incident T2D, we modelled serum Zn, Cu, and desaturase activities using restricted cubic splines (3 knots at the 10th, 50th, 90th percentiles) and assessed departure from linearity with likelihood-ratio tests comparing spline models against linear models.

To investigate if Zn or Cu status or the Cu/Zn ratio modify the associations of FA biomarkers with T2D risk, we first stratified analyses by serum Zn or Cu (<median vs ≥ median of the subcohort) and tested multiplicative interaction by including cross-product terms of the FA profiles (continuous) and Cu or Zn (binary) in the multivariable-adjusted model. Additionally, we evaluated continuous effect modification using interactions between FA biomarkers and restricted cubic splines of Zn or Cu (knots: 10th, 50th, 90th percentiles), with nonlinear interaction tested by likelihood-ratio tests comparing models with spline-interaction terms against models with only linear interaction terms.

To test if the associations of Zn or Cu and T2D are, in part, mediated by the desaturases, we conducted a mediation analysis. The attenuation of the association between Zn or Cu and T2D risk after adjustment for desaturase activities was evaluated by comparing Cox models without and with adjustment. The relative change of association and its stability, as well as the corresponding hazard ratios were estimated with a bootstrapping procedure (500 bootstrap replicates).

Additionally, to confirm a role of metal status in desaturase-T2D associations, we derived polygenic risk scores (PGS) for circulating Cu and Zn (**Supplementary Methods**). We assessed if genetic variants modify the relationships between FA levels or desaturase ratios and T2D risk by including multiplicative interaction terms with FA variables (continuous) and either the PGS (higher vs. lower by cohort median) or the prespecified variant in *SLC30A8* (minor-allele carriers vs. major-allele homozygotes). We then conducted stratified analyses based on the PGS or genotype of the selected SNP.

P-values below 0.05 were considered significant. All statistical analyses were performed using SAS software (version 9.4, Enterprise Guide 7.1).

## 3 Results

### 3.1 Baseline characteristics

The baseline characteristics of the EPIC-Potsdam subcohort participants according to Zn and Cu status are shown in Table 1. Median serum Zn levels were 724 μg/L (IQR = 636–820 μg/L). The proportion of women was highest in the lowest Zn quartile (69%). Higher Zn levels were related to higher median BMI and higher waist circumference. Participants in the highest Zn quartile also tended to have higher alcohol intake and Cu levels, and lower HDL-cholesterol levels. The estimated D5D and SCD1-18 activities appeared to be lower in individuals with higher Zn levels. Serum Cu levels had a median of 1016 μg/L (IQR = 877-1200 μg/L). Participants with higher Cu concentrations were more likely women (34% Q1 versus 92% Q4). Those in the highest Cu quartile also tended to have lower median waist circumference, alcohol intake, and triglyceride levels, higher HDL-cholesterol levels, and lower estimated D5D.

**Table 1:** Baseline characteristics according to quartiles of serum zinc (Zn) and copper (Cu) in the EPIC-Potsdam random subcohort (n = 1577).

	Quartiles of serum Zn			
Serum Zn (μg/L)	Q1	Q2	Q3	Q4
	585 (547–612)	682 (662–703)	766 (745–789)	909 (857–987)
Age (years)	47.5 (40.9–56.7)	49.9 (42.8–57.7)	51.6 (41.9–58.3)	49 (42.1–57.7)
Sex (% women)	69.4	61.2	61.3	59.5
Body mass index (kg/m^2^)	24.7 (22.7–27.7)	25.6 (23.1–28.4)	25.6 (23.1–28.1)	26.1 (23.4–28.8)
Waist circumference (cm)	82.0 (73.5–91.5)	85.0 (76.0–94.5)	84.0 (76.0–94.0)	87.0 (77.5–94.0)
Physical activity (hours/week)	2.0 (0.5–4.0)	1.5 (0.0–4.0)	2.0 (0.0–4.0)	1.5 (0.0–4.0)
Alcohol from alcoholic drinks (g/day)	7.3 (2.6–18.4)	8.9 (3.3–19.6)	8.0 (2.5–20.3)	8.9 (3.3–19.6)
Type of work (%)				
(Heavy) manual work	4.3	5.9	6.8	6.8
Sedentary occupation	63.8	59.1	58.2	60.0
Standing occupation	31.9	35.0	34.9	33.2
Education (%)				
No vocational or vocational training	34.9	41.7	34.7	37.5
Technical college	21.4	24.4	28.4	22.8
High education/university	43.7	33.9	37.0	39.8
Smoking status (%)				
Never smoking	49.8	41.7	45.6	49.6
Ex-smoker	29.9	36.5	31.1	31.1
Smoker <20 units/day	14.3	14.1	17.0	14.2
Smoker ≥20 units/day	6.0	7.7	6.3	5.1
Cu (μg/L)	1008 (865–1216)	1019 (868–1172)	1012 (873–1184)	1031 (893–1266)
Fe (μg/L)	812 (647–1018)	880 (696–1087)	980 (756–1190)	1034 (837–1279)
Triglycerides (mg/dL)	102.3 (73.2–162.8)	112.8 (74.4–167.4)	105.8 (74.1–153.5)	107.0 (76.7–153.5)
Total cholesterol (mg/dL)	202.1 (176.7–227.7)	204.6 (180.2–230.1)	211.6 (181.4–234.8)	207.8 (180.8–236)
HDL-cholesterol (mg/dL)	57.3 (48.3–66.6)	55.5 (46.8–64.6)	56 (48.2–66.2)	54.4 (46.5–63.3)
D5D (20:4n-6/20:3n-6)^a^	8.9 (7.5–10.4)	8.8 (7.5–10.1)	8.8 (7.6–10.1)	8.7 (7.4–9.9)
D6D (18:3n-6/18:2n-6)^a^	0.005 (0.003–0.006)	0.005 (0.003–0.007)	0.005 (0.003–0.006)	0.005 (0.003–0.007)
SCD1-16 (16:1n-7/16:0)^a^	0.019 (0.016–0.024)	0.019 (0.015–0.024)	0.02 (0.016–0.026)	0.02 (0.015–0.025)
SCD1-18 (18:1n-9/18:0)^a^	0.95 (0.86–1.05)	0.92 (0.86–1.01)	0.93 (0.86–1.01)	0.90 (0.84–0.99)

	Quartiles of serum Cu			
Serum Cu (μg/L)	Q1	Q2	Q3	Q4
	795 (721–836)	943 (907–979)	1096 (1055–1143)	1428 (1294–1669)
Age (years)	48.6 (42.6–56.7)	51.6 (42.5–58.8)	52.8 (44.0–59.1)	45.6 (39.3–55.1)
Sex (% women)	34.0	51.4	74.4	91.6
BMI (kg/m^2^)	25.2 (23.3–27.8)	25.6 (23.0–28.1)	26.2 (23.5–29.3)	24.9 (22.6–28.2)
Waist circumference (cm)	88.0 (78.0–95.0)	87.0 (76.5–96.0)	84.5 (76.5–93.0)	79.8 (73–89)
Physical activity (h/week)	2.0 (0.5–4.0)	2.0 (0.0–4.5)	1.5 (0.0–4.0)	1.5 (0.0–4.0)
Alcohol from alcoholic drinks (g/day)	10.4 (3.7–23.2)	9.9 (2.9–22.8)	7.1 (2.2–17.9)	6.1 (2.5–11.4)
Type of work (%)				
(Heavy) manual work	7.9	9.1	5.3	1.5
Sedentary occupation	59.1	61.5	59.6	60.9
Standing occupation	33.0	29.4	35.0	37.6
Education (%)				
No vocational or vocational training	29.4	41.0	39.9	38.3
Technical college	18.8	20.8	28.7	28.7
High education/university	51.8	38.2	31.5	33.0
Smoking status (%)				
Never smoking	38.6	43.8	53.8	50.5
Ex-smoker	40.4	32.7	26.1	29.4
Smoker <20 units/day	16.0	16.5	13.7	13.5
Smoker ≥20 units/day	5.0	7.1	6.4	6.6
Zn (μg/L)	713 (624–792)	738 (642–831)	718 (642–806)	731 (636–846)
Fe (μg/L)	908 (713–1170)	943 (737–1134)	891 (722–1083)	947 (711–1219)
Triglycerides (mg/dL)	113.3 (76.7–179.6)	107.0 (74.4–167.9)	103.5 (70.9–148.2)	102.3 (75.6–143.0)
Total cholesterol (mg/dL)	201.9 (175–226.7)	209.3 (181.4–238.3)	208.6 (183.7–232.5)	203.4 (177.9–229.0)
HDL-cholesterol (mg/dL)	52.0 (43.9–61.7)	53.7 (46.9–64.5)	57.0 (48.8–65.6)	59.4 (49.7–69.6)
D5D (20:4n-6/20:3n-6)^a^	8.8 (7.7–10.1)	9.0 (7.6–10.3)	9.0 (7.7–10.2)	8.5 (7.3–9.6)
D6D (18:3n-6/18:2n-6)^a^	0.005 (0.003–0.007)	0.005 (0.003–0.007)	0.005 (0.004–0.007)	0.004 (0.003–0.006)
SCD1-16 (16:1n-7/16:0)^a^	0.02 (0.015–0.023)	0.02 (0.015–0.025)	0.02 (0.015–0.025)	0.02 (0.017–0.026)
SCD1-18 (18:1n-9/18:0)^a^	0.93 (0.86–1.03)	0.92 (0.85–1.01)	0.92 (0.86–1.01)	0.92 (0.86–1.02)

Data are median (IQR) for continuous measures and % for categorical measures.

a Product-to-precursor fatty acid ratios were used to estimate desaturase enzyme activities: delta-5 desaturase (D5D), delta-6 desaturase (D6D), and stearoyl-CoA desaturase-1 (SCD1).

### 3.2 Associations between Zn, Cu or Fe and estimated desaturase activities or fatty acids

**Fig. 1:**
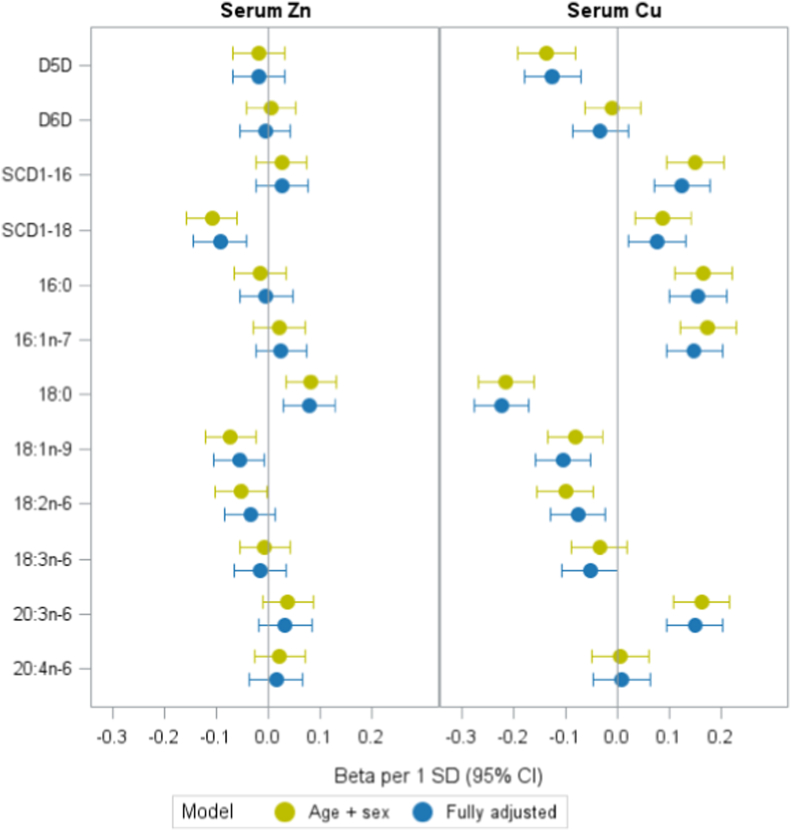
**Associations of serum Zinc (Zn) and Copper (Cu) with estimated desaturase activities and related fatty acids in the EPIC-Potsdam random subcohort (n=1577)** Estimates were derived from linear regression models adjusted for age and sex and the fully adjusted model was adjusted for age, sex, waist circumference, height, leisure-time physical activity, highest achieved education level, smoking status, alcohol intake, and fasting status. Both exposures (Zn, Cu) and outcomes (desaturase ratios and fatty acids) were log-transformed and Z-standardized. Estimated desaturase activities were calculated for delta-5 desaturase (D5D) = 20:4n-6/20:3n-6; delta-6 desaturase (D6D) = 18:3n-6/18:2n-6 and stearoyl-CoA desaturase-1 (SCD1): SCD1-16 = 16:1n-7/16:0; SCD1-18 = 18:1n-9/18:0.

In the subcohort, we examined the associations between Zn and Cu levels with estimated desaturase activities, along with their FA substrates and products, adjusting for potential confounders (Fig. 1). Zn levels were inversely associated with the estimated activity of SCD1-18 (18:1n-9/18:0) (β per 1-SD higher Zn = -0.09, p < 0.001). Cu was slightly negatively associated with estimated D5D activity (β = -0.13, p < 0.001), which was driven by a positive association of Cu with the precursor FA 20:3n-6 (β = 0.15, p < 0.001). Cu was positively associated with SCD1-16 (β = 0.13, p < 0.001) and SCD1-18 activity (β = 0.08, p = 0.005). Fe was associated with SCD1-16 (β = 0.07, p = 0.004) and, consistently, with 16:1n-7 (β = 0.07, p = 0.003), but not with 16:0 (β = 0.03, p = 0.198) (Fig. S2).

### 3.3 Associations of desaturase activities or fatty acids with T2D incidence according to Zn and Cu serum levels and the Cu/Zn ratio

**Fig. 2:**
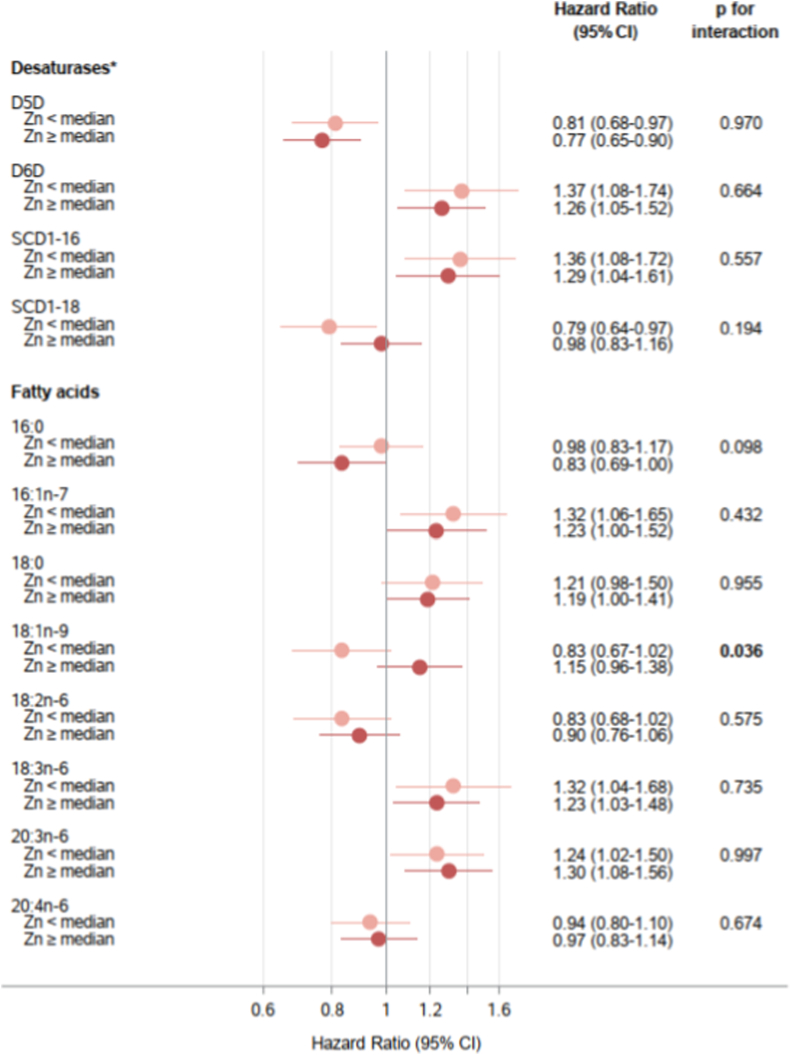
**Associations of estimated desaturase activities ^a^ and related fatty acids with type 2 diabetes incidence stratified by serum zinc (Zn) levels (below and above median 724 μg/L)**, EPIC-Potsdam study Hazard ratio and confidence interval (CI) per 1 SD difference of each fatty acid or desaturase ratio (n = 1979 including subcohort = 1577 and cases = 447). Models were adjusted for age (as underlying time variable), sex, waist circumference, height, leisure-time physical activity, highest achieved education level, smoking status, alcohol intake, and fasting status at blood draw. Tests for statistical interactions were conducted by including cross-product terms of desaturase activities or fatty acids (continuous) and serum Zn (binary: below/above median) in the models. ^a^ Estimated desaturase activities were calculated for delta-5 desaturase (D5D) = 20:4n-6/20:3n-6; delta-6 desaturase (D6D) = 18:3n-6/18:2n-6 and stearoyl-CoA desaturase-1 (SCD1): SCD1-16 = 16:1n-7/16:0; SCD1-18 = 18:1n-9/18:0.

We first examined if there were any non-linear associations of serum Zn, Cu, and each desaturase with incident T2D using restricted cubic splines. We found no evidence of nonlinearity for Zn, Cu, or any desaturase ratio (all p for nonlinearity >0.05) (Fig. S3). Next, we evaluated the associations of estimated desaturase activities and individual fatty acids with T2D after stratifying participants by Zn status (above and below the median) using models adjusted for demographic, lifestyle, and anthropometric risk factors (Fig. 2).

Significant interactions were observed between Zn status and 18:1n-9 (p-interaction = 0.036). In participants with lower Zn levels, 18:1n-9 exhibited an inverse association with T2D risk (HR per SD = 0.83, 95% CI 0.67–1.02), while this association was positive in those with higher Zn levels (HR = 1.15, 95% CI 0.96–1.38). While the inverse association of estimated SCD1-18 activity with T2D risk appeared to be stronger in participants with lower Zn levels (HR = 0.79, 95% CI 0.64–0.97) compared to those with higher Zn levels (HR = 0.98, 95% CI 0.83–1.16), there was no statistically significant interaction. We did not observe marked differences in T2D associations across Zn status subgroups for other FA biomarkers (all p-interaction >0.05). Further adjusting the models for blood lipids, medication, serum Cu and serum Fe did not markedly impact these associations (Table S2). When testing for non-linear interactions with restricted cubic splines, we found a significant non-linear Zn and D5D interaction (p = 0.03). However, consistent with the stratified analyses, the estimated D5D activity was inversely associated with T2D across the range of Zn concentrations (Fig. S4). No other FA biomarker showed evidence of non-linear interaction with Zn (all p > 0.05).

**Fig. 3:**
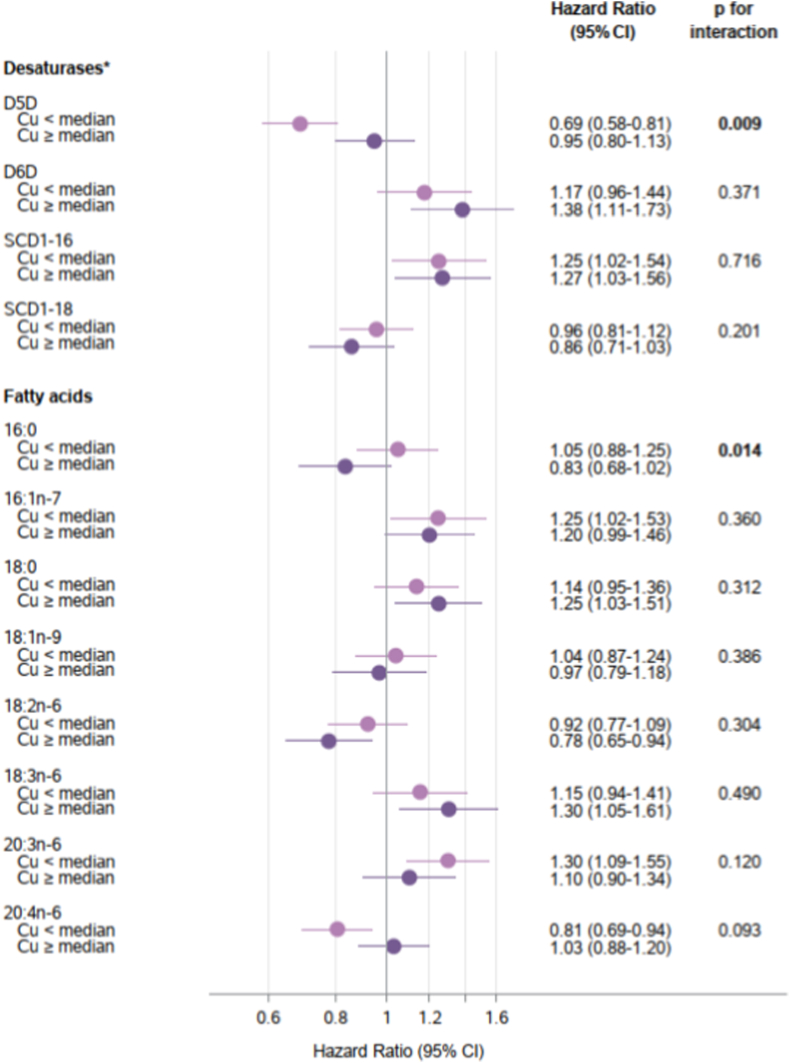
**Associations of estimated desaturase activities ^a^ and related fatty acids with type 2 diabetes incidence stratified by serum copper (Cu) levels (below and above median of 1016 μg/L)**, EPIC-Potsdam study Hazard ratio and confidence interval (CI) per 1 SD difference of each fatty acid or desaturase ratio (n = 1979 including subcohort = 1577 and cases = 447). Models were adjusted for age (as underlying time variable), sex, waist circumference, height, leisure-time physical activity, highest achieved education level, smoking status, alcohol intake, and fasting status at blood draw. Tests for statistical interactions were conducted by including cross-product terms of desaturase activities or fatty acids (continuous) and serum Cu (binary: below/above median) in the models. ^a^ Estimated desaturase activities were calculated for delta-5 desaturase (D5D) = 20:4n-6/20:3n-6; delta-6 desaturase (D6D) = 18:3n-6/18:2n-6 and stearoyl-CoA desaturase-1 (SCD1): SCD1-16 = 16:1n-7/16:0; SCD1-18 = 18:1n-9/18:0.

Significant interactions were observed between Cu status and D5D (p-interaction = 0.009). A significant inverse association between estimated D5D activity and T2D risk was observed among participants with lower serum Cu levels (HR = 0.69, 95% CI 0.58–0.81). The association of D5D was weaker and not statistically significant in participants with Cu levels above the median (HR = 0.95, 95% CI 0.80–1.13) (Fig. 3). Although the association of D6D activity with T2D appeared to be more pronounced at high Cu levels, we did not detect statistically significant interactions. Statistical interaction was not detectable for SCD1 ratios or individual FAs, except 16:0, which showed an inverse association with T2D at higher Cu levels (HR = 0.83, 95% CI 0.68–1.02) but not at lower levels (HR = 1.05, 95% CI 0.88–1.25) (p-interaction = 0.014). Further adjusting the models for blood lipids, medication, serum Zn, and serum Fe did not significantly impact these associations (Table S3). We identified a significant nonlinear Cu and D5D interaction (p = 0.01). Consistent with stratified analyses, the protective D5D-T2D association was strongest at low Cu concentrations and weaker at higher Cu levels (Fig. S5). Other fatty acid biomarkers showed no nonlinear interactions with Cu (all p > 0.05). Furthermore, because of substantial differences in Cu levels between women and men, as a sensitivity analysis, we stratified the models by sex. In both men an women, the protective association of D5D was statistically significant only below the overall Cu median of 1016 μg/L, however, the interaction term was statistically significant only in men (Table S4).

We observed a significant interaction between the Cu/Zn ratio and estimated SCD1-18 activity in relation to T2D risk (p-interaction = 0.004) (Fig. S6). Among participants with Cu/Zn ratios above the median, higher SCD1-18 activity was significantly associated with reduced T2D risk (HR = 0.74, 95% CI 0.61–0.90), whereas no association was observed in those with ratios below the median (HR = 1.05, 95% CI 0.90–1.23). Investigating single FA components of SCD1-18, the Cu/Zn ratio modified the associations of 18:1n-9 with T2D (p-interaction = 0.010), but not of 18:0. No other significant interactions were observed between the Cu/Zn ratio and remaining desaturase activities or individual fatty acids.

### 3.4 Mediation analyses

Higher serum Zn and Cu were associated with higher T2D risk (HR per 1 SD: 1.18, 95% CI 1.10–1.32 and 1.12, 1.02–1.22) and mediation analysis suggested that desaturase activities partly explained these associations (Table 2). Estimated D5D accounted for 9.6% of the Zn-T2D association (% change of HR: -9.6, 95% CI -21.8% to -3.6%) and -20.5% (% change of HR: -20.5, 95% CI -93.6% to -5.8%) of the Cu-T2D association. Similarly, SCD1-16 mediated 18.6% of the Cu and T2D relationship (% change of HR: -18.6, 95% CI -72.6% to -4.9%).

**Table 2:** Mediation analysis of associations of zinc and copper with type 2 diabetes by desaturases, EPIC-Potsdam study (total n = 1979; cases n = 447**)**.

	Zinc		Copper	
	HR (95% CI)^a^	% change (95% CI)^b^	HR (95% CI)^a^	% change (95% CI)^b^
Reference model	1.18 (1.10, 1.32)		1.12 (1.02, 1.22)	
+ D5D	1.16 (1.08, 1.28)	**-9.6 (-21.8, -3.6)**	1.09 (1.00, 1.19)	**-20.5 (-93.6, -5.8)**
+ D6D	1.18 (1.11, 1.34)	1.8 (-2.5, 9.8)	1.13 (1.03, 1.24)	9.2 (-1.54, 45.7)
+ SCD1-16	1.17 (1.10, 1.31)	-1.8 (-7.4, 3.1)	1.09 (1.00, 1.20)	**-18.6 (-72.6, -4.9)**
+ SCD1-18	1.17 (1.10, 1.31)	-4.3(-11.2, 0.5)	1.12 (1.02, 1.22)	0.6 (-7.7, 7.6)

a Hazard ratio (HR) and 95% confidence interval (CI) per 1-SD difference of serum zinc or copper concentrations, estimated as median and dispersion from a bootstrapping procedure (500 bootstrap replicates). Models adjusted for age (as underlying time variable), sex, waist circumference, height, leisure-time physical activity, highest achieved education level, smoking status, alcohol intake, and fasting status at blood draw.

b The % change reflects the estimated change of HRs after additional adjustment for each estimated desaturase activity, relative to the HR estimate from the reference model. Its stability as well as the corresponding HR were estimated as median and dispersion from a bootstrapping procedure (500 bootstrap replicates).

### 3.5 Association of FA biomarkers with T2D risk in strata according to Zn- and Cu-related genetic variants

The Zn-related PGS and the individual variants (rs2120019, rs1532423, rs322884) were not associated with serum Zn in our subcohort (Table S5). Although we found no evidence of interactions between the Zn-PGS and desaturases (Table 3), the association between 20:3n-6 and T2D risk was stronger in participants with higher compared to lower Zn-PGS (p-interaction = 0.004). The Zn-related *SLC30A8* SNP rs13266634 modified the association of estimated D5D activity with T2D risk (p-interaction = 0.014): a stronger inverse association was observed in individuals with the CT- or TT-genotypes (HR = 0.66, 95% CI 0.55–0.78) compared to those with the CC-genotype (HR = 0.91, 95% CI 0.77–1.08) (Table 3). This finding was supported by a stronger positive association between 20:3n-6 levels and T2D risk in participants with CT- or TT-genotypes (HR = 1.65, 95% CI 1.35–2.02) compared with the CC-genotype (HR = 1.03, 95% CI 0.85–1.25) (p-interaction = 0.001).

**Table 3:** Association of estimated desaturase activities and single fatty acids with type 2 diabetes risk, stratified by polygenic score (PGS) related to serum Zinc (Zn)^a^ and genotypes of a SNPs related to Zn transporter ZnT8^b^, EPIC-Potsdam study.

Desaturase/Fatty acid	HR per 1-SD (95% CI)^c^		p for interaction^d^	HR per 1-SD (95% CI)^c^ by rs13266634 genotype (*SLC30A8)*		p for interaction^d^
	Lower PGS	Higher PGS		CC	CT + TT	
D5D (20:4n-6/20:3n-6)	0.93 (0.73–1.19)	0.73 (0.63–0.85)	0.127	**0.91 (0.77–1.08)**	**0.66 (0.55–0.78)**	**0.014**
D6D (18:3n-6/18:2n-6)	1.65 (1.21–2.24)	1.17 (0.96–1.41)	0.192	1.51 (1.21–1.88)	1.22 (1.00–1.48)	0.125
SCD1-16 (16:1n-7/16:0)	1.23 (0.91–1.67)	1.38 (1.13–1.69)	0.402	1.46 (1.19–1.78)	1.25 (0.99–1.58)	0.442
SCD1-18 (18:1n-9/18:0)	0.78 (0.63–0.98)	0.95 (0.80–1.12)	0.244	0.91 (0.75–1.10)	0.90 (0.75–1.08)	0.812
16:0	0.86 (0.66–1.11)	0.90 (0.75–1.06)	0.791	0.87 (0.70–1.08)	0.91 (0.77–1.08)	0.701
16.1n-7	1.18 (0.88–1.60)	1.32 (1.09–1.60)	0.442	1.39 (1.14–1.68)	1.21 (0.96–1.51)	0.482
18:0	1.36 (1.07–1.73)	1.14 (0.96–1.36)	0.216	1.41 (1.12–1.78)	1.11 (0.93–1.33)	0.160
18:1n-9	0.93 (0.72–1.20)	1.04 (0.87–1.23)	0.700	1.11 (0.92–1.35)	0.95 (0.78–1.16)	0.201
18:2n-6	0.88 (0.68–1.14)	0.86 (0.73–1.01)	0.389	0.76 (0.63–0.92)	0.95 (0.78–1.15)	0.125
18:3n-6	1.64 (1.21–2.24)	1.11 (0.92–1.34)	0.069	1.40 (1.13–1.74)	1.21 (0.99–1.48)	0.244
20:3n-6	**0.91 (0.72–1.16)**	**1.50 (1.26–1.78)**	**0.004**	**1.03 (0.85–1.25)**	**1.65 (1.35–2.02)**	**0.001**
20:4n-6	0.83 (0.68–1.01)	1.00 (0.86–1.15)	0.193	0.91 (0.75–1.11)	0.96 (0.83–1.10)	0.699

a Participants with complete SNPs data for PGS (total n = 1818; subcohort n = 1457; incident cases n = 403) were stratified by median PGS into lower score (n = 611; cases n = 121; median serum Zn = 735 μg/L) and higher score (n = 1207; cases n = 282; median serum Zn = 730 μg/L).

b Participants with SNP rs13266634 data (total n = 1890; subcohort = 1577; incident cases = 424) were stratified by *SLC30A8* rs13266634 genotype into CC (n total = 889, cases = 203, median serum Zn = 733 μg/L) and CT + TT (n total = 1001, cases = 221, median serum Zn = 731 μg/L).

c Hazard ratios (HR) and 95% confidence intervals (95% CI) derived from a multivariable Cox regression. Models were adjusted for age (as underlying time variable), sex, waist circumference, height, leisure-time physical activity, highest achieved education level, smoking status, alcohol intake, and fasting status at blood draw. Product-to-precursor fatty acid ratios were used to estimate desaturase enzyme activities: delta5 desaturase (D5D), delta6 desaturase (D6D), and stearoyl-CoA desaturase-1 (SCD1).

d Derived from tests for statistical interactions of fatty acid profile and desaturase activities (estimated by erythrocyte fatty acid ratios) with Zn-related PGS (binary) or Zn-related SNP-genotype (binary) by including cross-product terms in the models.

We also examined genetic variation related to circulating Cu. The Cu-PGS was modestly associated with serum Cu levels and, among individual Cu variants (rs2769264, rs17564336, rs1175550, rs3858704, rs10424895, rs34004251), only rs34004251 was associated with serum Cu in the subcohort (Table S5). The inverse association of D5D with T2D was stronger in participants with higher Cu-PGS than lower PGS (p-interaction = 0.029) (Table 4).

**Table 4:** Associations of desaturase activities and single fatty acids with type 2 diabetes risk, stratified by polygenic score (PGS) related to serum copper (Cu) levels^a^, EPIC-Potsdam study.

	HR per 1-SD (95% CI)^b^		p for interaction^c^
	Lower PGS	Higher PGS	
D5D (20:4n-6/20:3n-6)	**0.87 (0.72–1.05)**	**0.59 (0.48–0.73)**	**0.029**
D6D (18:3n-6/18:2n-6)	1.41 (1.09–1.81)	1.30 (1.01–1.67)	0.528
SCD1-16 (16:1n-7/16:0)	1.32 (1.04–1.66)	1.40 (1.04–1.88)	0.946
SCD1-18 (18:1n-9/18:0)	0.82 (0.68–0.99)	1.16 (0.94–1.43)	0.067
16:0	0.89 (0.73–1.08)	1.08 (0.86–1.37)	0.308
16.1n-7	1.26 (1.01–1.57)	1.41 (1.04–1.91)	0.718
18:0	1.29 (1.05–1.60)	0.99 (0.78–1.26)	0.189
18:1n-9	0.92 (0.75–1.13)	1.24 (1.00–1.54)	0.152
18:2n-6	0.92 (0.75–1.12)	1.01 (0.76–1.35)	0.301
18:3n-6	1.40 (1.09–1.80)	1.30 (1.02–1.67)	0.652
20:3n-6	1.37 (1.08–1.72)	1.52 (1.21–1.92)	0.609
20:4n-6	1.10 (0.93–1.32)	0.82 (0.68–0.98)	0.116

a Participants with complete SNPs data for PGS (total n = 1526; subcohort n = 1228; incident cases n = 298) were stratified by median PGS into lower score (n = 873; cases n = 188; median serum Cu = 1009 μg/L) and higher score (n = 653; cases n = 143; median serum Cu = 1087 μg/L).

b Hazard ratios (HR) and 95% confidence intervals (95% CI) derived from a multivariable Cox regression. Models were adjusted for age (as underlying time variable), sex, waist circumference, height, leisure-time physical activity, highest achieved education level, smoking status, alcohol intake, and fasting status at blood draw. Product-to-precursor fatty acid ratios were used to estimate desaturase enzyme activities: delta-5 desaturase (D5D), delta-6 desaturase (D6D), and stearoyl-CoA desaturase-1 (SCD1).

c Derived from tests for statistical interactions of fatty acid levels and desaturase activities (estimated by erythrocyte fatty acid ratios) with Cu-related PGS (binary) by including cross-product terms in the models.

## 4 Discussion

In this prospective cohort study, our primary aim was to examine whether circulating Zn and Cu, trace elements involved in redox homeostasis, insulin signaling, and shown to influence lipid desaturation, modulate estimated desaturase activities (SCD1-16, SCD1-18, D5D, D6D) and their association with T2D risk. We found that Zn levels were negatively associated with SCD1 activity, while Cu levels were positively associated with SCD1 activity and negatively associated with D5D activity. In addition, we observed effect modification by the trace element status: Zn levels modified the association of D5D and 18:1n-9 with T2D risk, and Cu levels modified the relationship between D5D activity and 16:0 with T2D risk. Genetic variation involved in Zn-transport pathways and genetic variants related to circulating Cu both modified the association between D5D and T2D. In mediation analyses, D5D and SCD1-16 accounted for approximately 20% of the association of Cu with T2D. These findings suggest an interplay between the trace element status and its genetic regulation, FA desaturation pathways, and T2D risk.

We observed that higher serum Zn concentrations were associated with lower SCD1 activity (estimated as 18:1n-9/18:0). These findings align with those from a controlled feeding study in men, where correcting Zn deficiency through supplementation, which increased plasma Zn levels, led to decreased estimated SCD1 activity [39]. Furthermore, our findings suggest that at lower Zn concentrations, higher SCD1 activity and its product, 18:1n-9, are inversely associated with T2D risk. Inconsistent associations between 18:1n-9 and T2D have been previously reported across various cohorts [40], which may reflect unmeasured modifiers such as Zn status. Conditions of Zn deficiency can increase insulin resistance and inflammation, while 18:1n-9 has been shown to improve insulin sensitivity and exert anti-inflammatory effects in cell culture experiments [41,42].

Although we observed a significant non-linear interaction between Zn and D5D activity, the association of D5D with T2D remained protective across Zn levels, indicating any effect modification by serum Zn is modest. Previous data involving 2189 men, however, linked higher serum Zn concentrations with lower estimated D5D activity and, although they did not find statistically significant interactions, the inverse associations between D5D and T2D were stronger below the Zn median [13]. Furthermore, because circulating Zn is homeostatically regulated, it may not completely capture other Zn pools relevant for desaturase regulation. Supporting this, in controlled feeding studies, Zn-fortified foods decreased D5D activity, despite unchanged plasma Zn levels [39,43]. In individuals with T2D, Zn supplementation increased gene expression of *FADS1* (gene encoding for the D5D enzyme), with no change in *FADS2* expression (encoding D6D) [44], supporting a link between Zn and D5D rather than D6D.

To gain insights into potential mechanisms independent of circulating Zn, we explored whether a genetic variant affecting Zn transport in β-cells modified desaturase and T2D associations. The *SLC30A8* variant rs13266634, modified the D5D-T2D association in our study. This SNP has been consistently associated with T2D risk [25,26,45]. *SLC30A8* encodes Zn transporter ZnT8, which impacts β-cell Zn homeostasis and insulin metabolism [24,46], providing a plausible mechanism by which altered intracellular Zn handling in β-cells influences insulin secretion and hepatic clearance [47], which in turn modulates hepatic lipogenesis and regulation of D5D activity.

Our findings also suggest a pathway linking Cu with T2D through FA metabolism. We found a negative association between serum Cu levels and estimated D5D activity, and D5D partly accounted for the Cu association with T2D. Human evidence linking Cu depletion with D5D activity exists (indicated by elevated 20:4n-6 with low Cu intake) [18] and animal studies showed that low Cu diets increased D5D activity (evidenced by elevated 20:4n-6 and reduced 20:3n-6 in plasma and liver) [16]. Notably, we found that the association of D5D with T2D risk varied across the range of serum Cu in a non-linear manner. Consistent with our observations of serum Cu levels, we found an interaction between D5D and Cu-PGS with T2D risk. A mechanistic explanation of the link of Cu-D5D-T2D is that lower Cu increases hepatic SREBP-1 activation [48], which transcriptionally upregulates *FADS1* (D5D) expression [49], favoring the conversion of 20:3n-6 to 20:4n-6 and thereby reducing circulating 20:3n-6 levels, which are associated with increased T2D risk [3]. Studies consistently show that women have significantly higher plasma/serum Cu concentrations than men [[50], [51], [52]]. These differences are primarily attributed to estrogen-mediated increases in ceruloplasmin [51,52]. The sex-specific patterns we observed suggest that Cu-desaturase-T2D relationships may have threshold effects and operate through different mechanisms in men and women, potentially related to sex differences in Cu metabolism. Future studies specifically designed to investigate sex-specific thresholds and mechanisms are needed to fully characterize these relationships.

We also found evidence of the link between Cu with SCD1. Besides the positive associations between serum Cu and SCD1-16 and SCD1-18, SCD1-16 explained about 20% of the Cu with T2D association in our study. Previous human evidence of the link between Cu and SCD1 is limited. Small studies in men indicated that plasma Cu levels correlated positively with 18:1n-9 [53] and that low-Cu diets decreased serum 18:1n-9 [18]. In rats, Cu deficiency markedly reduced the desaturation of ^14^C-labeled 18:0 to 18:1n-9 when measured directly in liver microsomes [17], and was associated with a lower estimated SCD1-18 activity [16,17,54]. For SCD1-16, Cu supplementation led to higher 16:1n-7 and lower 16:0 levels [16]. Altered Cu status can modulate SCD1 via oxidative stress-mediated activation of SREBP-1 in hepatocytes [55], which upregulates SCD1 gene expression [56]. SCD1 activity influences lipogenesis, lipid signaling, and insulin sensitivity, all relevant to T2D [57].

A limitation of our study is the use of FA ratios as proxies for desaturase activity, rather than direct enzymatic measurements. Additionally, our analyses were based on single baseline measurements of Zn, Cu, and FA, which limits our ability to assess the stability of these measures over time and may result in misclassification due to random error potentially underestimating true associations. Although we adjusted for various confounders, the potential for residual confounding remains. The limited validation of the PGS against serum levels in our cohort restricts confidence in genetic instruments, and tissue-specific Zn or Cu may not be captured by circulating levels. We also acknowledge the potential for false-positive findings due to multiple testing. Despite these limitations, our study has significant strengths. The prospective design, extensive follow-up, large number of events, and low loss to follow-up enhance the reliability of our findings. Importantly, this is one of the first studies to prospectively investigate the interactions between Zn, Cu, genetic variants, and desaturase activities on T2D risk in a large human cohort, offering novel insights into how these minerals may influence FA metabolism and its metabolic consequences.

In conclusion, this study highlights the potential roles of Zn and Cu in influencing T2D risk through their interaction with desaturase enzyme activities and FA metabolism. Circulating levels of Cu and Zn modified the associations of desaturase activities with T2D risk. We provide evidence that serum Cu significantly modifies the D5D-T2D association, with validation using genetically predicted circulating Cu levels. Furthermore, the association of Cu levels with T2D risk is partially explained by desaturase activities. Understanding how the status of these trace elements and their genetic regulation can modify the relationship between desaturase activities and T2D risk may guide the development of tailored nutrition interventions as a precision prevention approach for T2D.

## Funding

This work was supported by the Deutsche Forschungsgemeinschaft (DFG, German Research Foundation) for TraceAge (DFG Research Unit on Interactions of essential trace elements in healthy and diseased elderly, Potsdam-Berlin-Jena, FOR 255, SCHU 1516/6-1, SCHU 1516/6-2, HA 4318/4-2). The EPIC-Potsdam recruitment phase was supported by the Federal Ministry of Science, Germany (Grant 01 EA 9401) and the European Union (Grant SOC 95201408 05 F02). Follow-up of EPIC-Potsdam was supported by the German Cancer Aid (Grant 70–2488-Ha I) and the European Community (Grant SOC 98200769 05 F02). This work was furthermore supported by a grant from the German Federal Ministry of Research, Technology and Space (BMFTR) to the German Center for Diabetes Research (DZD) (82DZD03D03) and the State of Brandenburg.

## CRediT authorship contribution statement

**Marcela Prada:** Conceptualization, Data curation, Formal analysis, Investigation, Methodology, Visualization, Writing – original draft, Writing – review & editing. **Olha Bezuhla:** Conceptualization, Data curation, Formal analysis, Writing – original draft. **Olga Kuxhaus:** Data curation, Formal analysis, Methodology, Writing – original draft. **Fabian Eichelmann:** Methodology, Visualization, Writing – original draft, Writing – review & editing. **Susanne Jäger:** Conceptualization, Data curation, Writing – review & editing. **Anna P. Kipp:** Investigation, Methodology, Writing – review & editing. **Hajo Haase:** Investigation, Methodology, Writing – review & editing. **Tanja Schwerdtle:** Formal analysis, Investigation, Writing – review & editing. **Matthias B. Schulze:** Conceptualization, Funding acquisition, Investigation, Methodology, Project administration, Resources, Supervision, Writing – review & editing.

## Declaration of competing interest

The authors declare that they have no known competing financial interests or personal relationships that could have appeared to influence the work reported in this paper.

## Data Availability

The datasets analysed during the current study are not publicly available due to data protection regulations. In accordance with German Federal and State data protection regulations, epidemiological data analyses of EPIC-Potsdam may be initiated upon an informal inquiry addressed to the secretariat of the Human Study Center (office.hsz@dife.de). Each request will then have to pass a formal process of application and review by the respective principal investigator and a scientific board.
