# Supplementary material for: Interaction of serum zinc and copper status with fatty acid desaturases on incidence of type 2 diabetes in the EPIC-Potsdam study
Source: Redox Biol. 2025 Dec 26;90:103998. doi: 10.1016/j.redox.2025.103998 (PMC12818309; doi:10.1016/j.redox.2025.103998)
Supplement: Multimedia component 1 [file mmc1.docx]

**SUPPLEMENTARY MATERIAL**

**Interaction of serum zinc and copper status with fatty acid desaturases on incidence of type 2 diabetes in the** **EPIC-Potsdam cohort study**

Marcela Prada, Olha Bezuhla, Olga Kuxhaus, Fabian Eichelmann, Susanne Jäger, Anna P. Kipp, Hajo Haase, Tanja Schwerdtle, Matthias B. Schulze

Contents

[Supplementary Methods: Genetic Risk Scores 3](#_Toc215514298)

[Table S1. Genetic variants related to Zinc (Zn) or Copper (Cu) 4](#_Toc215514299)

[Table S2. Association between desaturase activities and single fatty acids and type 2 diabetes risk by serum zinc (Zn) level, EPIC-Potsdam case-cohort (n = 1979 ^a^) 5](#_Toc215514300)

[Table S3. Association between desaturase activities and single fatty acids and type 2 diabetes by serum copper (Cu) level, EPIC-Potsdam case-cohort (n=1,979 ^a^) 7](#_Toc215514301)

[Table S4. Association between desaturase activities and single fatty acids and type 2 diabetes by serum copper (Cu) level, EPIC-Potsdam case-cohort (n=1,979) stratified by subcohort serum Cu median (1016 µg/L) and sex 9](#_Toc215514302)

[Table S5. Associations of single candidate SNPs and polygenic risk scores (PGS) with serum zinc (Zn) and serum copper (Cu), EPIC-Potsdam subcohort 11](#_Toc215514303)

[Figure S1. Study population 12](#_Toc215514304)

[Figure S2. Associations of serum iron (Fe) with estimated desaturase activities ^a^ and related fatty acids in the EPIC-Potsdam subcohort (n = 1577) ^b^ 13](#_Toc215514305)

[Figure S3. Associations of zinc (Zn), copper (Cu) and desaturases with type 2 diabetes risk using restricted cubic splines, EPIC-Potsdam case-cohort 14](#_Toc215514306)

[Figure S4. Non-linear associations between estimated delta-5 desaturase (D5D) activity and type 2 diabetes risk across serum zinc (Zn) levels, EPIC-Potsdam case-cohort 15](#_Toc215514307)

[Figure S5. Non-linear associations between estimated delta-5 desaturase (D5D) activity and type 2 diabetes risk across serum copper (Cu) levels, EPIC-Potsdam case-cohort 16](#_Toc215514308)

[Figure S6. Associations of estimated desaturase activities and related fatty acids with type 2 diabetes incidence stratified by the ratio of serum copper to zinc (Cu/Zn) (below and above median of 1.42), EPIC-Potsdam case-cohort 17](#_Toc215514309)

[References 18](#_Toc215514310)

# Supplementary Methods: Genetic Risk Scores

We identified SNPs related to circulating Zn and Cu levels from published GWAS (1-4), using the NHGRI-EBI GWAS Catalog and a literature search for recent references. We included loci reaching genome-wide significance (p < 5×10^-8^) in the discovery Genome-Wide Association Studies (GWAS). We selected those available in our data and linkage disequilibrium (LD) independent (r² < 0.8) (reference panel: 1000 Genomes, European). We retained only one representative SNP from each LD block, prioritizing variants with the strongest association evidence (largest effect sizes from the original GWAS).

The Cu-related candidates were rs2769264 (2), rs17564336 (3), rs1175550 (2), rs3858704 (4), rs10424895(4), rs34004251 (1). The Zn-related candidates were rs2120019 (2), rs1532423 (2), rs322884 (1). For each SNP, we aligned genotype dosages to the metal‑increasing allele as reported in the discovery GWAS.

Because published SNP effects were reported on incompatible scales (log and rank‑normalized), we constructed metal‑specific unweighted polygenic risk scores (PGS). Participants with missing allele dosage at any included SNP were excluded from the score calculation. We then summed metal‑increasing allele dosages across SNPs.

For analyses, the PGS was dichotomized at the subcohort median to define low vs high groups. We validated the zinc-related PGS (Zn-PGS) and the copper-related PGS (Cu-PGS) in the subcohort using linear regression models adjusted for age and sex.

Effect modification was assessed with multiplicative interaction terms between continuous desaturase and fatty acid variables and the binary PGS (high vs low). We also estimated stratum‑specific associations by fitting models within PGS strata. Case–cohort Cox models used Prentice weights with robust variance and adjusted for multiple variables.

# Table S1. Genetic variants related to Zinc (Zn) or Copper (Cu)

| **SNP ID** | | **Gene** | **Effect allele ^a^** | **Other allele** | **Effect allele frequency ^b^** | **Sample size ^b^** | **Effect ^a^** |
| --- | --- | --- | --- | --- | --- | --- | --- |
|  | |  |  |  |  |  |  |
| rs13266634 | | *SLC30A8* | C | T | 0.69 | 1525 | Higher T2D risk |
| rs2120019 | | *PPCDC* | T | C | 0.79 | 1519 | Higher Zn levels |
| rs1532423 | | *CA1* | A | G | 0.39 | 1523 | Higher Zn levels |
| rs322884 | | *LINC01221* | A | C | 0.30 | 1467 | Higher Zn levels |
| rs2769264 | | *SELENBP1* | G | T | 0.18 | 1521 | Higher Cu levels |
| rs34004251 | | *CP* | A | T | 0.16 | 1401 | Higher Cu levels |
| rs1175550 | | *SMIM1* | G | A | 0.22 | 1371 | Higher Cu levels |
| rs10424895 | | *OR7C1* | A | G | 0.62 | 1519 | Higher Cu levels |
| rs3858704 | | *CUX2* | G | A | 0.25 | 1526 | Higher Cu levels |
| rs17564336 | | *SELENBP1,PSMB4* | G | T | 0.71 | 1481 | Higher Cu levels |
|  |  | |  |  |  |  |  |

^a^ Effect direction (higher Cu, Zn, or T2D risk) was based on discovery Genome-Wide Association Studies (GWAS) (1-5). Effect alleles in this table were harmonized to represent the allele associated with increasing the respective trait (T2D risk, Zn or Cu levels)

^b^ In the EPIC-Potsdam subcohort

# Table S2. Association between desaturase activities and single fatty acids and type 2 diabetes risk by serum zinc (Zn) level, EPIC-Potsdam case-cohort (n = 1979 ^a^)

|  | **Serum Zn level below median (< 724 µg/L)** | **Serum Zn level above median (≥ 724 µg/L)** |
| --- | --- | --- |
|  | **Hazard Ratio per 1-SD (95% Confidence Interval)** ^b^ | |
| **D5D (20:4n-6/20:3n-6)** |  |  |
| Model 1 | 0.75 (0.65-0.87) | 0.68 (0.59-0.78) |
| Model 2 | 0.81 (0.68-0.97) | 0.77 (0.65-0.90) |
| Model 3 | 0.83 (0.69-1.00) | 0.83 (0.70-0.98) |
| Model 4 | 0.81 (0.68-0.97) | 0.77 (0.65-0.91) |
| Model 5 | 0.81 (0.68-0.97) | 0.77 (0.66-0.91) |
| **D6D (18:3n-6/18:2n-6)** |  |  |
| Model 1 | 1.55 (1.24-1.94) | 1.49 (1.27-1.75) |
| Model 2 | 1.37 (1.08-1.74) | 1.26 (1.05-1.52) |
| Model 3 | 1.31 (1.01-1.70) | 1.21 (1.00-1.46) |
| Model 4 | 1.38 (1.08-1.75) | 1.27 (1.05-1.52) |
| Model 5 | 1.37 (1.08-1.74) | 1.26 (1.05-1.51) |
| **SCD1-16 (16:1n-7/16:0)** |  |  |
| Model 1 | 1.51 (1.22-1.87) | 1.50 (1.23-1.82) |
| Model 2 | 1.36 (1.08-1.72) | 1.29 (1.04-1.61) |
| Model 3 | 1.40 (1.09-1.80) | 1.18 (0.96-1.46) |
| Model 4 | 1.36 (1.08-1.72) | 1.29 (1.04-1.60) |
| Model 5 | 1.36 (1.08-1.72) | 1.28 (1.03-1.58) |
| **SCD1-18 (18:1n-9/18:0)** |  |  |
| Model 1 | 0.89 (0.74-1.06) | 1.08 (0.93-1.25) |
| Model 2 | 0.79 (0.64-0.97) | 0.98 (0.83-1.16) |
| Model 3 | 0.75 (0.61-0.93) | 0.85 (0.72-1.00) |
| Model 4 | 0.79 (0.64-0.97) | 0.98 (0.83-1.16) |
| Model 5 | 0.79 (0.65-0.97) | 0.98 (0.83-1.16) |
| **16:0 (Palmitic acid)** |  |  |
| Model 1 | 1.02 (0.89-1.18) | 0.93 (0.79-1.10) |
| Model 2 | 0.98 (0.83-1.17) | 0.83 (0.69-1.00) |
| Model 3 | 1.02 (0.85-1.22) | 0.85 (0.71-1.03) |
| Model 4 | 0.98 (0.82-1.16) | 0.83 (0.69-1.00) |
| Model 5 | 0.98 (0.83-1.17) | 0.83 (0.69-1.00) |
| **16:1n-7 (Palmitoleic acid)** | |  |
| Model 1 | 1.47 (1.20-1.79) | 1.44 (1.19-1.75) |
| Model 2 | 1.32 (1.06-1.65) | 1.23 (1.00-1.52) |
| Model 3 | 1.38 (1.08-1.75) | 1.14 (0.92-1.40) |
| Model 4 | 1.32 (1.06-1.65) | 1.23 (1.00-1.51) |
| Model 5 | 1.32 (1.06-1.65) | 1.22 (0.99-1.50) |
| **18:0 (Stearic acid)** |  |  |
| Model 1 | 1.21 (1.01-1.45) | 1.17 (1.01-1.35) |
| Model 2 | 1.21 (0.98-1.50) | 1.19 (1.00-1.41) |
| Model 3 | 1.19 (0.96-1.47) | 1.21 (1.02-1.43) |
| Model 4 | 1.22 (0.99-1.50) | 1.21 (1.01-1.44) |
| Model 5 | 1.21 (0.98-1.50) | 1.19 (1.01-1.41) |
| **18:1n-9 (Oleic acid)** |  |  |
| Model 1 | 0.99 (0.82-1.18) | 1.26 (1.08-1.48) |
| Model 2 | 0.83 (0.67-1.02) | 1.15 (0.96-1.38) |
| Model 3 | 0.77 (0.62-0.95) | 0.95 (0.78-1.15) |
| Model 4 | 0.83 (0.68-1.03) | 1.16 (0.97-1.39) |
| Model 5 | 0.83 (0.68-1.02) | 1.15 (0.96-1.38) |
| **18:2n-6 (Linoleic acid)** |  |  |
| Model 1 | 0.77 (0.65-0.91) | 0.80 (0.69-0.92) |
| Model 2 | 0.83 (0.68-1.02) | 0.90 (0.76-1.06) |
| Model 3 | 0.85 (0.69-1.04) | 0.91 (0.76-1.08) |
| Model 4 | 0.84 (0.68-1.02) | 0.90 (0.76-1.07) |
| Model 5 | 0.83 (0.68-1.02) | 0.90 (0.76-1.07) |
| **18:3n-6 (γ-Linoleic acid)** |  |  |
| Model 1 | 1.47 (1.17-1.85) | 1.45 (1.23-1.70) |
| Model 2 | 1.32 (1.04-1.68) | 1.23 (1.03-1.48) |
| Model 3 | 1.25 (0.97-1.63) | 1.19 (0.98-1.43) |
| Model 4 | 1.33 (1.04-1.70) | 1.24 (1.04-1.49) |
| Model 5 | 1.32 (1.04-1.68) | 1.23 (1.03-1.48) |
| **20:3n-6 (Dihomo-γ-linolenic acid)** | |  |
| Model 1 | 1.38 (1.17-1.62) | 1.44 (1.23-1.68) |
| Model 2 | 1.24 (1.02-1.50) | 1.30 (1.08-1.56) |
| Model 3 | 1.18 (0.96-1.45) | 1.21 (1.01-1.46) |
| Model 4 | 1.23 (1.02-1.50) | 1.29 (1.08-1.55) |
| Model 5 | 1.24 (1.02-1.50) | 1.30 (1.08-1.56) |
| **20:4n-6 (Arachidonic acid)** | |  |
| Model 1 | 0.95 (0.82-1.10) | 0.92 (0.82-1.04) |
| Model 2 | 0.94 (0.80-1.10) | 0.97 (0.83-1.14) |
| Model 3 | 0.93 (0.78-1.10) | 0.98 (0.83-1.16) |
| Model 4 | 0.94 (0.80-1.10) | 0.97 (0.83-1.14) |
| Model 5 | 0.94 (0.80-1.11) | 0.97 (0.83-1.13) |

^a^ Below the median were 937 participants (including 170 incident diabetes cases) and above the median 1042 participants (including 277 incident diabetes cases)

^b^ Hazard ratios per 1 standard deviation higher concentration, derived from models adjusted for:

**Model 1**: age (as underlying time variable), sex,
**Model 2**: Model 1 and waist circumference, height, leisure-time physical activity (hours/week), highest achieved education level (in or no training, skilled worker, technical school, or university degree), smoking status (never, past, current, <20 cigarettes/day, or current >20 cigarettes/day), alcohol intake (0, 0.1–5.0, 5.1–10.0, 10.1–20.0, 20.1–40.0, or >40.0 g/day), fasting status at blood draw

**Model 3**: Model 2 and total cholesterol, HDL-cholesterol, triglycerides, antihypertensive medication, lipid-lowering medication, acetylsalicylic acid medication
**Model 4**: Model 2 and serum copper (µg/L)

**Model 5**: Model 2 and serum iron (µg/L)

# Table S3. Association between desaturase activities and single fatty acids and type 2 diabetes by serum copper (Cu) level, EPIC-Potsdam case-cohort (n=1,979 ^a^)

|  | **Serum Cu level below median (< 1016 µg/L)** | **Serum Cu level above median (****≥ 1016 µg/L)** |
| --- | --- | --- |
|  | **Hazard Ratio per 1-SD (95% CI)** ^b^ | |
| **D5D (20:4n-6/20:3n-6)** |  |  |
| Model 1 | 0.66 (0.57-0.76) | 0.77 (0.67-0.88) |
| Model 2 | 0.69 (0.58-0.81) | 0.95 (0.80-1.13) |
| Model 3 | 0.72 (0.60-0.86) | 0.96 (0.80-1.15) |
| Model 4 | 0.69 (0.58-0.81) | 0.95 (0.80-1.13) |
| Model 5 | 0.69 (0.58-0.81) | 0.95 (0.80-1.13) |
| **D6D (18:3n-6/18:2n-6)** |  |  |
| Model 1 | 1.46 (1.24-1.72) | 1.59 (1.28-1.97) |
| Model 2 | 1.17 (0.96-1.44) | 1.38 (1.11-1.73) |
| Model 3 | 1.14 (0.92-1.41) | 1.41 (1.11-1.80) |
| Model 4 | 1.17 (0.96-1.43) | 1.41 (1.13-1.76) |
| Model 5 | 1.18 (0.96-1.44) | 1.38 (1.10-1.72) |
| **SCD1-16 (16:1n-7/16:0)** |  |  |
| Model 1 | 1.48 (1.23-1.78) | 1.46 (1.19-1.80) |
| Model 2 | 1.25 (1.02-1.54) | 1.27 (1.03-1.56) |
| Model 3 | 1.17 (0.94-1.44) | 1.26 (1.01-1.56) |
| Model 4 | 1.25 (1.02-1.54) | 1.26 (1.03-1.55) |
| Model 5 | 1.25 (1.02-1.54) | 1.26 (1.02-1.54) |
| **SCD1-18 (18:1n-9/18:0)** |  |  |
| Model 1 | 1.01 (0.88-1.16) | 0.94 (0.78-1.13) |
| Model 2 | 0.96 (0.81-1.12) | 0.86 (0.71-1.03) |
| Model 3 | 0.83 (0.71-0.98) | 0.76 (0.63-0.93) |
| Model 4 | 0.96 (0.82-1.12) | 0.87 (0.72-1.04) |
| Model 5 | 0.95 (0.81-1.12) | 0.86 (0.71-1.03) |
| **16:0 (Palmitic acid)** |  |  |
| Model 1 | 1.05 (0.91-1.22) | 0.88 (0.74-1.04) |
| Model 2 | 1.05 (0.88-1.25) | 0.83 (0.68-1.02) |
| Model 3 | 1.02 (0.86-1.22) | 0.85 (0.69-1.04) |
| Model 4 | 1.05 (0.88-1.25) | 0.83 (0.68-1.01) |
| Model 5 | 1.05 (0.88-1.25) | 0.83 (0.67-1.01) |
| **16:1n-7 (Palmitoleic acid)** | |  |
| Model 1 | 1.47 (1.22-1.77) | 1.38 (1.13-1.68) |
| Model 2 | 1.25 (1.02-1.53) | 1.20 (0.99-1.46) |
| Model 3 | 1.16 (0.94-1.44) | 1.19 (0.97-1.46) |
| Model 4 | 1.25 (1.01-1.53) | 1.19 (0.98-1.45) |
| Model 5 | 1.25 (1.02-1.54) | 1.19 (0.98-1.44) |
| **18:0 (Stearic acid)** |  |  |
| Model 1 | 1.16 (1.00-1.35) | 1.25 (1.05-1.48) |
| Model 2 | 1.14 (0.95-1.36) | 1.25 (1.03-1.51) |
| Model 3 | 1.16 (0.96-1.40) | 1.33 (1.09-1.61) |
| Model 4 | 1.13 (0.94-1.36) | 1.24 (1.03-1.50) |
| Model 5 | 1.14 (0.95-1.36) | 1.25 (1.04-1.51) |
| **18:1n-9 (Oleic acid)** |  |  |
| Model 1 | 1.15 (0.99-1.33) | 1.10 (0.91-1.33) |
| Model 2 | 1.04 (0.87-1.24) | 0.97 (0.79-1.18) |
| Model 3 | 0.86 (0.72-1.03) | 0.87 (0.70-1.09) |
| Model 4 | 1.04 (0.87-1.24) | 0.98 (0.80-1.19) |
| Model 5 | 1.04 (0.87-1.24) | 0.97 (0.79-1.18) |
| **18:2n-6 (Linoleic acid)** |  |  |
| Model 1 | 0.82 (0.72-0.94) | 0.71 (0.60-0.84) |
| Model 2 | 0.92 (0.77-1.09) | 0.78 (0.65-0.94) |
| Model 3 | 0.92 (0.77-1.09) | 0.75 (0.62-0.91) |
| Model 4 | 0.92 (0.78-1.09) | 0.78 (0.65-0.94) |
| Model 5 | 0.92 (0.77-1.09) | 0.78 (0.65-0.95) |
| **18:3n-6 (γ-Linoleic acid)** |  |  |
| Model 1 | 1.43 (1.21-1.71) | 1.47 (1.20-1.81) |
| Model 2 | 1.15 (0.94-1.41) | 1.30 (1.05-1.61) |
| Model 3 | 1.11 (0.90-1.38) | 1.30 (1.04-1.64) |
| Model 4 | 1.15 (0.94-1.41) | 1.33 (1.07-1.64) |
| Model 5 | 1.15 (0.94-1.42) | 1.30 (1.05-1.61) |
| **20:3n-6 (Dihomo-γ-linolenic acid)** | |  |
| Model 1 | 1.42 (1.22-1.65) | 1.42 (1.21-1.67) |
| Model 2 | 1.30 (1.09-1.55) | 1.10 (0.90-1.34) |
| Model 3 | 1.24 (1.02-1.50) | 1.08 (0.87-1.35) |
| Model 4 | 1.30 (1.09-1.55) | 1.10 (0.90-1.35) |
| Model 5 | 1.30 (1.09-1.55) | 1.10 (0.90-1.34) |
| **20:4n-6 (Arachidonic acid)** | |  |
| Model 1 | 0.84 (0.73-0.97) | 1.03 (0.91-1.17) |
| Model 2 | 0.81 (0.69-0.94) | 1.03 (0.88-1.20) |
| Model 3 | 0.83 (0.71-0.97) | 1.03 (0.86-1.22) |
| Model 4 | 0.81 (0.69-0.94) | 1.04 (0.89-1.21) |
| Model 5 | 0.81 (0.69-0.94) | 1.03 (0.88-1.20) |

^a^ Below the median were 1013 participants (including 248 incident diabetes cases) and above the median 966 participants (including 199 incident diabetes cases)

^b^ Hazard ratios per 1 standard deviation higher concentration, derived from models adjusted for:

**Model 1**: age (as underlying time variable), sex,
**Model 2**: Model 1 and waist circumference, height, leisure-time physical activity (hours/week), highest achieved education level (in or no training, skilled worker, technical school, or university degree), smoking status (never, past, current, <20 cigarettes/day, or current >20 cigarettes/day), alcohol intake (0, 0.1–5.0, 5.1–10.0, 10.1–20.0, 20.1–40.0, or >40.0 g/day), fasting status at blood draw

**Model 3**: Model 2 and total cholesterol, HDL-cholesterol, triglycerides, antihypertensive medication, lipid-lowering medication, acetylsalicylic acid medication
**Model 4**: Model 2 and serum zinc (µg/L)

**Model 5**: Model 2 and serum iron (µg/L)

# Table S4. Association between desaturase activities and single fatty acids and type 2 diabetes by serum copper (Cu) level, EPIC-Potsdam case-cohort (n=1,979) stratified by subcohort serum Cu median (1016 µg/L) and sex

|  |  | **Men^a^** | |  | **Women^b^** | |
| --- | --- | --- | --- | --- | --- | --- |
|  |  | **Hazard Ratio**  **(95% CI)** | **p-interaction** |  | **Hazard Ratio**  **(95% CI)** | **p-interaction** |
| **Desaturases** |  |  |  |  |  |  |
| **D5D** |  |  |  |  |  |  |
| Cu < median |  | 0.74 (0.60-0.90) | 0.025 |  | 0.71 (0.52-0.99) | 0.392 |
| Cu ≥ median |  | 1.10 (0.74-1.63) |  |  | 0.83 (0.68-1.01) |  |
| **D6D** |  |  |  |  |  |  |
| Cu < median |  | 1.01 (0.79-1.28) | 0.132 |  | 1.57 (1.08-2.27) | 0.574 |
| Cu ≥ median |  | 2.48 (1.35-4.54) |  |  | 1.43 (1.09-1.88) |  |
| **SCD1-16** |  |  |  |  |  |  |
| Cu < median |  | 1.22 (0.99-1.50) | 0.661 |  | 1.36 (1.02-1.82) | 0.833 |
| Cu ≥ median |  | 1.86 (1.00-3.47) |  |  | 1.32 (1.00-1.73) |  |
| **SCD1-18** |  |  |  |  |  |  |
| Cu < median |  | 0.93 (0.78-1.12) | 0.113 |  | 0.92 (0.67-1.26) | 0.940 |
| Cu ≥ median |  | 0.83 (0.48-1.44) |  |  | 0.87 (0.68-1.12) |  |
|  |  |  |  |  |  |  |
| **Fatty acids** |  |  |  |  |  |  |
| **16:00** |  |  |  |  |  |  |
| Cu < median |  | 0.90 (0.74-1.11) | 0.006 |  | 1.65 (1.24-2.19) | 0.017 |
| Cu ≥ median |  | 0.55 (0.32-0.96) |  |  | 0.91 (0.73-1.13) |  |
| **16:1n-7** |  |  |  |  |  |  |
| Cu < median |  | 1.18 (0.96-1.46) | 0.232 |  | 1.45 (1.06-1.99) | 0.541 |
| Cu ≥ median |  | 1.59 (0.95-2.65) |  |  | 1.24 (0.97-1.60) |  |
| **18:00** |  |  |  |  |  |  |
| Cu < median |  | 1.12 (0.91-1.39) | 0.534 |  | 1.06 (0.74-1.52) | 0.765 |
| Cu ≥ median |  | 1.21 (0.66-2.21) |  |  | 1.35 (1.07-1.70) |  |
| **18:1n-9** |  |  |  |  |  |  |
| Cu < median |  | 1.00 (0.82-1.21) | 0.181 |  | 0.93 (0.68-1.27) | 0.999 |
| Cu ≥ median |  | 0.88 (0.46-1.67) |  |  | 1.07 (0.84-1.37) |  |
| **18:2n-6** |  |  |  |  |  |  |
| Cu < median |  | 0.96 (0.79-1.17) | 0.156 |  | 0.63 (0.43-0.93) | 0.363 |
| Cu ≥ median |  | 0.48 (0.30-0.75) |  |  | 0.77 (0.61-0.96) |  |
| **18:3n-6** |  |  |  |  |  |  |
| Cu < median |  | 0.99 (0.78-1.26) | 0.169 |  | 1.43 (0.98-2.10) | 0.678 |
| Cu ≥ median |  | 2.06 (1.10-3.84) |  |  | 1.34 (1.03-1.75) |  |
| **20:3n-6** |  |  |  |  |  |  |
| Cu < median |  | 1.28 (1.04-1.57) | 0.438 |  | 1.05 (0.72-1.53) | 0.812 |
| Cu ≥ median |  | 1.15 (0.76-1.74) |  |  | 1.27 (0.99-1.62) |  |
| **20:4n-6** |  |  |  |  |  |  |
| Cu < median |  | 0.90 (0.76-1.07) | 0.085 |  | 0.72 (0.56-0.93) | 0.102 |
| Cu ≥ median |  | 1.20 (0.81-1.80) |  |  | 1.00 (0.82-1.21) |  |

^a^ Men: 621 participants (184 incident T2D cases) had Cu <1016 µg/L and 195 participants (66 incident T2D cases) had Cu ≥1016 µg/L.
^b^ Women: 392 participants (64 incident T2D cases) had Cu <1016 µg/L and 771 participants (133 incident T2D cases) had Cu ≥1016 µg/L.

Hazard ratios per 1 standard deviation higher concentration, derived from models adjusted for age (as underlying time variable), sex, waist circumference, height, leisure-time physical activity (hours/week), highest achieved education level (in or no training, skilled worker, technical school, or university degree), smoking status (never, past, current, <20 cigarettes/day, or current >20 cigarettes/day), alcohol intake (0, 0.1–5.0, 5.1–10.0, 10.1–20.0, 20.1–40.0, or >40.0 g/day), fasting status at blood draw

Tests for statistical interactions were conducted by including cross-product terms of desaturase activities or fatty acids (continuous) and Cu/Zn ratio (binary: below/above subcohort median 1016 µg/L) in the models.

Estimated desaturase activities were calculated for delta-5 desaturase (D5D) = 20:4n-6/20:3n-6; delta-6 desaturase (D6D) = 18:3n-6/18:2n-6 and stearoyl-CoA desaturase-1 (SCD1): SCD1-16= 16:1n-7/16:0; SCD1-18=18:1n-9/18:0

# Table S5. Associations of single candidate SNPs and polygenic risk scores (PGS) with serum zinc (Zn) and serum copper (Cu), EPIC-Potsdam subcohort

|  | **Beta coefficient** | **Standard Error** | **t Value** | **p Value** |
| --- | --- | --- | --- | --- |
| **Associations with Zn (µg/L)** |  |  |  |  |
| PGS-Zn ^a^ | 0.44 | 6.15 | 0.07 | 0.940 |
| rs2120019 ^b^ | 14.67 | 12.13 | 1.21 | 0.227 |
| rs1532423 ^b^ | -8.25 | 9.93 | -0.83 | 0.406 |
| rs322884 ^b^ | -0.45 | 10.45 | -0.04 | 0.964 |
| **Associations with Cu (µg/L)** |  |  |  |  |
| PGS-Cu ^a^ | 12.48 | 5.43 | 2.30 | 0.022 |
| rs2769264 ^b^ | 4.82 | 15.33 | 0.31 | 0.753 |
| rs34004251 ^b^ | 61.94 | 15.73 | 3.94 | <.0001 |
| rs1175550 ^b^ | -15.10 | 14.33 | -1.05 | 0.292 |
| rs10424895 ^b^ | 8.30 | 12.00 | 0.69 | 0.489 |
| rs3858704 ^b^ | 19.09 | 13.53 | 1.41 | 0.159 |
| rs17564336 ^b^ | 10.82 | 12.81 | 0.84 | 0.399 |

^a^ Associations evaluated using linear regression models adjusted for age and sex, The genetic risk scores were calculated for subcohort participants with complete data for Zn-PGS (n=1457) or Cu-PGS (n=1228)

^b^ Linear regression under an additive genetic model, coding each SNP as 0/1/2 copies of the effect allele and adjusting for age and sex. The beta coefficient estimates the change in Zn or Cu per additional effect allele

EPIC-Potsdam cohort

**n = 27548**

Exclusion of participants without blood samples

EPIC-Potsdam cohort

**n = 26437**

Incident type 2 diabetes cases

**n = 801**

Random subcohort

**n = 2500**

Exclusions

Incident type 2 diabetes cases

**n = 447**

Random subcohort

**n = 1577**

Total

**n = 1979**

Overlapping

**n = 74**

missing serum Zn and Cu (n=297)

unreliable fatty acid measurements (n=833)

missing follow-up information (n=36)

prevalent or non-verifiable cases (n=72)

missing covariables (n=10)

Overlapping

**n = 45**

# Figure S1. Study population

The study population was derived from a random subcohort (n = 2500) and all individuals who developed type 2 diabetes during follow-up (n = 801). After exclusions, the final study population consisted of 1577 participants from the random subcohort and 447 participants with incident type 2 diabetes, resulting in a total of 1979 participants for the main analyses.

For genetic variant analyses, data were available for 1890 case-cohort participants. This included 1510 participants from the random subcohort and 424 participants with incident type 2 diabetes (including 44 cases within the subcohort).


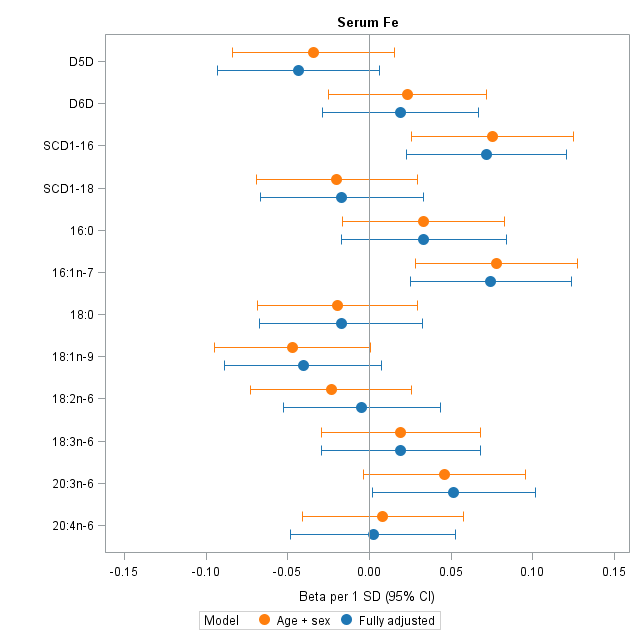


# Figure S2. Associations of serum iron (Fe) with estimated desaturase activities ^a^ and related fatty acids in the EPIC-Potsdam subcohort (n = 1577) ^b^

^a^ Estimated desaturase activities were calculated for delta-5 desaturase (D5D) = 20:4n-6/20:3n-6; delta-6 desaturase (D6D) = 18:3n-6/18:2n-6 and stearoyl-CoA desaturase-1 (SCD1): SCD1-16= 16:1n-7/16:0; SCD1-18=18:1n-9/18:0

^b^ Estimates were derived from linear regression modes adjusted for age and sex and fully adjusted for age, sex, waist circumference, height, leisure-time physical activity, highest achieved education level, smoking status, alcohol intake, and fasting status. Both exposure (Fe) and outcome (desaturase ratios and fatty acids) were log-transformed and Z-standardized.


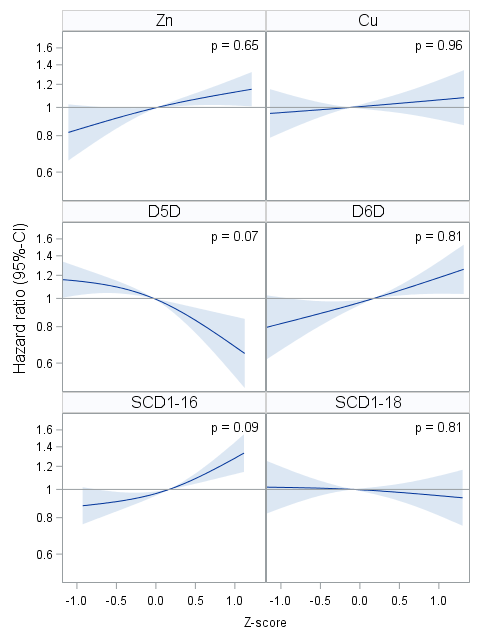


# Figure S3. Associations of zinc (Zn), copper (Cu) and desaturases with type 2 diabetes risk using restricted cubic splines, EPIC-Potsdam case-cohort

To assess potential departures from linearity, we modelled serum Zn, Cu, and each desaturase using restricted cubic splines with four knots at the 10th, 50th, 90th percentiles. Nonlinearity was evaluated with likelihood ratio tests comparing the spline model with a model containing only a linear term. Analyses using restricted cubic splines provided no evidence of nonlinearity for the association with type 2 diabetes risk of Zn, Cu, or the desaturase ratios: all p-nonlinearity >0.05


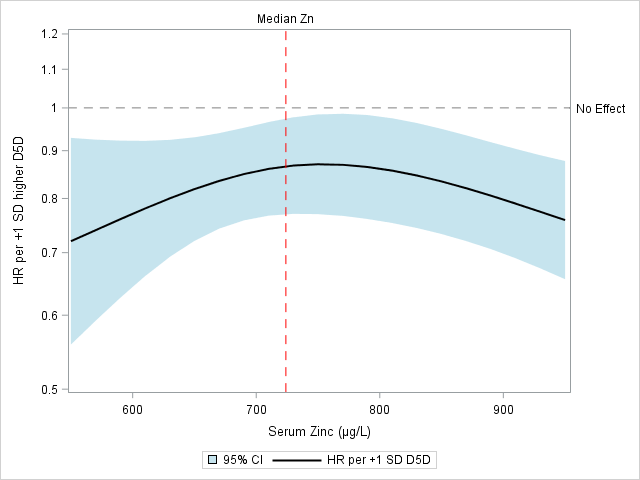


# Figure S4. Non-linear associations between estimated delta-5 desaturase (D5D) activity and type 2 diabetes risk across serum zinc (Zn) levels, EPIC-Potsdam case-cohort

Hazard ratios of estimated D5D activity (per 1 SD higher) and type 2 diabetes across serum zinc concentrations (μg/L). Associations were modelled using Cox proportional hazards regression with restricted cubic splines (3 knots at 10th, 50th, and 90th percentiles of zinc distribution). Models were adjusted for sex, waist circumference, height, physical activity, education, smoking status, occupational status, alcohol intake, and fasting status, with age as the time scale.


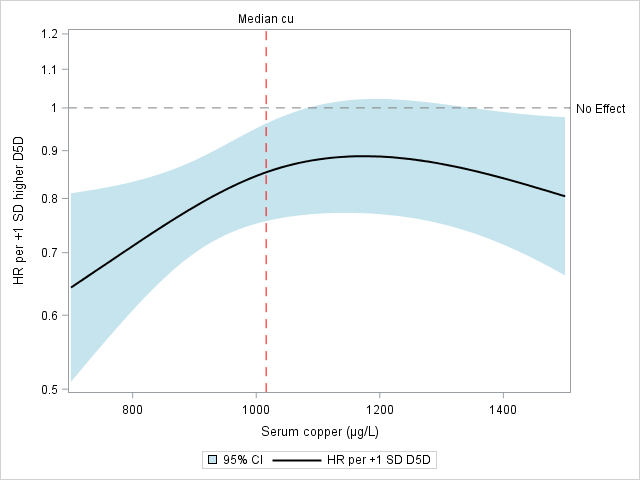


# Figure S5. Non-linear associations between estimated delta-5 desaturase (D5D) activity and type 2 diabetes risk across serum copper (Cu) levels, EPIC-Potsdam case-cohort

Hazard ratios of estimated D5D activity (per 1 SD higher) with type 2 diabetes across serum Cu concentrations (μg/L). Associations were modelled using Cox proportional hazards regression with restricted cubic splines (3 knots at 10th, 50th, and 90th percentiles of copper distribution). Models were adjusted for sex, waist circumference, height, physical activity, education, smoking status, occupational status, alcohol intake, and fasting status, with age as the time scale.


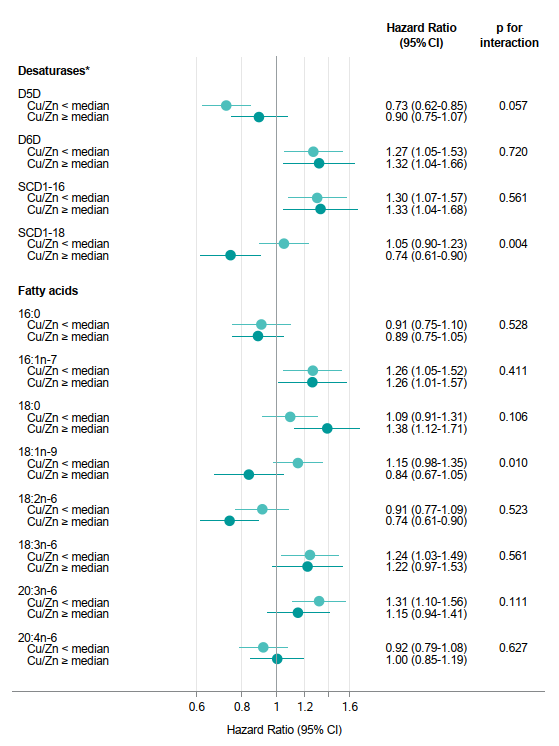


# Figure S6. Associations of estimated desaturase activities and related fatty acids with type 2 diabetes incidence stratified by the ratio of serum copper to zinc (Cu/Zn) (below and above median of 1.42), EPIC-Potsdam case-cohort

Hazard ratio and confidence interval (CI) per 1 SD increase of each fatty acid or desaturase ratio (n = 1979 including subcohort = 1577 and cases = 447). Models were adjusted for age (as underlying time variable), sex, waist circumference, height, leisure-time physical activity, highest achieved education level, smoking status, alcohol intake, and fasting status at blood draw. Tests for statistical interactions were conducted by including cross-product terms of desaturase activities or fatty acids (continuous) and Cu/Zn ratio (binary: below/above median) in the models.

Estimated desaturase activities were calculated for delta-5 desaturase (D5D) = 20:4n-6/20:3n-6; delta-6 desaturase (D6D) = 18:3n-6/18:2n-6 and stearoyl-CoA desaturase-1 (SCD1): SCD1-16= 16:1n-7/16:0; SCD1-18=18:1n-9/18:0

# References

1. Moksnes MR, Hansen AF, Wolford BN, Thomas LF, Rasheed H, Simić A, et al. A genome-wide association study provides insights into the genetic etiology of 57 essential and non-essential trace elements in humans. Communications Biology. 2024;7(1):432.

2. Evans DM, Zhu G, Dy V, Heath AC, Madden PA, Kemp JP, et al. Genome-wide association study identifies loci affecting blood copper, selenium and zinc. Human molecular genetics. 2013;22(19):3998-4006.

3. Jäger S, Cabral M, Kopp JF, Hoffmann P, Ng E, Whitfield JB, et al. Blood copper and risk of cardiometabolic diseases: a Mendelian randomization study. Hum Mol Genet. 2022;31(5):783-91.

4. Yang W, Li L, Feng X, Cheng H, Ge X, Bao Y, et al. Genome-wide association and Mendelian randomization study of blood copper levels and 213 deep phenotypes in humans. Commun Biol. 2022;5(1):405.

5. Sladek R, Rocheleau G, Rung J, Dina C, Shen L, Serre D, et al. A genome-wide association study identifies novel risk loci for type 2 diabetes. Nature. 2007;445(7130):881-5.
